# Integrated Service Delivery Models for Triple Elimination of Mother to Child Transmission of Human Immunodeficiency Virus, Syphilis, and Hepatitis B Virus: A Global Systematic Review and Meta-Analysis

**DOI:** 10.3390/healthcare14121625

**Published:** 2026-06-09

**Authors:** Victor Abiola Adepoju, Abdulrakib Abdulrahim, Qorinah Estiningtyas Sakilah Adnani, Shankar Biswas, Safayet Jamil, Uthman Okikiola Adebayo

**Affiliations:** 1 Department of HIV and Infectious Diseases, Jhpiego Nigeria (An Affiliate of Johns Hopkins University), Abuja 900911, Nigeria; 2 Department of Medical Microbiology, Faculty of Medicine and Health Sciences, Universiti Putra Malaysia, Serdang 43400, Malaysia; abdulrakib161@gmail.com; 3 Department of Public Health, Faculty of Medicine, Universitas Padjadjaran, Bandung 40161, Indonesia; qorinah.adnani@unpad.ac.id; 4 Department of Internal Medicine, Ivano–Frankivsk National Medical University, 76000 Ivano-Frankivsk, Ukraine; sb740927@gmail.com; 5 Department of Public Health, Daffodil International University, Dhaka 1216, Bangladesh; safayetkyau333@gmail.com; 6 Department of Public Health, Atish Dipankar University of Science and Technology, Dhaka 1230, Bangladesh; 7 Department of Medical Laboratory Science, Neuropsychiatric Hospital, Aro, Abeokuta 110101, Nigeria; uthmanadebayo85@gmail.com; 8 Department of Medical Laboratory Services, Federal University of Health Sciences Teaching Hospital, Ila-Orangun 234101, Nigeria; 9 Department of Digital Health, Global Health Focus Africa, Kigali 00000, Rwanda

**Keywords:** triple elimination of mother-to-child transmission, HIV, hepatitis B virus, syphilis, prenatal care, systematic review, meta-analysis

## Abstract

Background and Objectives: Despite global commitment to the World Health Organization triple elimination initiative, evidence on integrated antenatal service delivery models that simultaneously address human immunodeficiency virus (HIV), syphilis, and hepatitis B virus (HBV) remains fragmented, particularly across diverse health-system contexts. Eliminating vertical transmission of HIV, syphilis, and HBV is a global priority. Pregnant women are disproportionately affected by these infections, and untreated maternal disease leads to significant infant morbidity. Integrating antenatal screening and treatment provides an opportunity to address all three conditions simultaneously. Purpose: This systematic review and meta-analysis aimed to identify and synthesise evidence on integrated antenatal service delivery models addressing HIV, syphilis, and HBV simultaneously within maternal health services. It specifically examined model characteristics, screening uptake, treatment and follow-up outcomes, implementation barriers and facilitators, and evidence on cost-effectiveness. Methods: This systematic review and meta-analysis followed PRISMA 2020 guidelines and was registered in PROSPERO (CRD420261342186). We searched Scopus, PubMed, Web of Science, and Dimensions for studies published between January 2007 and January 2026. Of 423 records identified, 11 met the inclusion criteria after excluding two studies that did not provide empirical results for an integrated service model addressing all three target infections simultaneously. Data on study characteristics, service delivery, diagnostics, outcomes, and implementation factors were extracted. A random-effects meta-analysis of proportions was conducted using the DerSimonian–Laird estimator with logit transformation. Results: Eleven studies covered Asia, Africa, Europe, and Latin America, mostly in low- and lower-middle-income countries. Integration ranged from rapid test packages in community clinics to comprehensive programmes including STI treatment, malaria testing, and HBV birth-dose vaccination. Pooled triple testing uptake was 97% (95% CI 92 to 100%). Large programmes achieved over 99% coverage and reduced HIV vertical transmission to below 3%. Pilot studies showed feasibility but noted stockouts, data gaps, and weak treatment linkage. Economic analyses supported cost-effectiveness. Conclusions: Integrated antenatal services appear feasible and can achieve high testing uptake, particularly in well-supported programmes. However, evidence remains uneven regarding treatment completion, infant follow-up, HBV prophylaxis, long-term transmission outcomes, and sustainability in resource-constrained settings. Key challenges include supply constraints, workforce limitations, and follow-up gaps. Future research should evaluate the full care cascade, not screening uptake alone.

## 1. Introduction

Despite global commitment to the World Health Organization (WHO) triple elimination initiative, evidence on integrated antenatal service delivery models that simultaneously address human immunodeficiency virus (HIV), syphilis, and hepatitis B virus (HBV) remains fragmented [1,2,3]. Existing studies often focus on single infections, isolated screening outcomes, or high-income settings, leaving limited comparative evidence on implementation models, treatment linkage, infant follow-up, and health-system requirements [4]. This gap limits policymakers’ and programme managers’ ability to design integrated services that are effective, feasible, scalable, and sustainable across diverse health-system contexts.

Pregnant women and their infants bear a substantial burden of HIV, syphilis, and HBV infection. A recent systematic review estimated global pooled prevalence among pregnant women at 2.9% for HIV, 4.8% for HBV, and 0.8% for syphilis [1]. These aggregate figures mask significant inequalities. In low-income countries, prevalence increases to 5.2% for HIV, 6.6% for HBV, and 3.3% for syphilis [1]. Evidence from Ethiopia illustrates the magnitude of this burden, with prevalence rates of 7.6% for HIV, 4.5% for HBV, and 3.3% for syphilis among pregnant women, and dual infections occurring in up to 2% of cases [2]. If untreated, these infections can lead to high rates of vertical transmission and adverse pregnancy outcomes. Mother-to-child transmission (MTCT) occurs in approximately 15–45% of HIV infections, up to 90% among infants born to mothers with high HBV viral loads and positive hepatitis B e-antigen (HBeAg) status, and adverse outcomes in around 70% of untreated syphilis cases [3]. Beyond the direct health consequences, the long-term economic burden of caring for infected children places substantial strain on families and health systems, particularly in resource-constrained settings [3].

Recognising the interconnected nature of these infections, the WHO launched the Triple Elimination Initiative to support countries in eliminating mother-to-child transmission (EMTCT) of HIV, syphilis, and HBV [4,5]. The third edition of the WHO global guidance expanded earlier dual HIV and syphilis elimination efforts by incorporating HBV and outlining integrated intervention packages and monitoring indicators [6]. Countries are expected to achieve impact targets, including less than 2% HIV MTCT, fewer than 50 congenital syphilis cases per 100,000 live births, and less than 0.1% HBsAg prevalence among children aged five years. They must also sustain programme indicators, including at least 95% antenatal testing coverage, 95% treatment uptake among infected mothers, and 90% birth-dose HBV vaccination coverage [6].

Integrating testing and treatment for these infections within a single antenatal visit offers important logistical, economic, and health advantages [5]. Multiplex or combined rapid diagnostic tests (RDTs) enable simultaneous screening, reducing clinic visits, travel costs, and missed diagnostic opportunities. Economic modelling supports the value of this approach. In Cambodia, integrating triple screening was projected to reduce MTCT rates to 6.1% for HIV, 13% for HBV, and 4.6% for syphilis, with cost-effectiveness ranging from US$64 to US$114 per disability-adjusted life year (DALY) averted [7]. Similarly, microsimulation modelling in Nepal predicted that triple screening would avert 8166 DALYs compared with dual screening, at an incremental cost of US$114 per DALY averted [8].

Some countries have achieved significant progress in EMTCT [5]. China’s national integrated PMTCT programme expanded rapid testing for HIV, syphilis, and HBV from 5.5 million pregnant women in 2010 to 13.1 million in 2013, achieving over 99% testing coverage and reducing HIV MTCT to 6.7% [9]. Similarly, the Netherlands, where universal antenatal screening and HBV vaccination are implemented, reported screening coverage above 99% with no new MTCT cases [10].

However, substantial gaps persist in many regions. Antenatal care (ANC) coverage in sub-Saharan Africa remains limited. Syphilis screening coverage often lags behind HIV testing by 5–52% in several African countries, while HBV testing remains inconsistent [4]. Brazilian surveillance data reveal that syphilis rates increased sharply from 4.7 to 27.1 per 1000 live births between 2011 and 2021, accompanied by declining HBV vaccination coverage among infants from 96% to 76% [11]. Multiple barriers hinder implementation, including frequent stockouts of diagnostic tests and medications, limited availability of WHO prequalified triple RDTs, and workforce constraints [7]. Socio-cultural factors including stigma and limited awareness further reduce service uptake [12]. The epidemiological and programmatic dimensions of this burden are substantial. Global burden-of-disease estimates indicate that in 2021, over 1.5 million women of reproductive age were living with HIV and over 100 million with chronic HBV infection, with disproportionate concentrations in sub-Saharan Africa and South and Southeast Asia [3]. In sub-Saharan Africa, where the triple burden is most acute, elimination targets for HIV, syphilis, and HBV remain far from reach, with syphilis screening coverage lagging behind HIV testing by wide margins in multiple countries and HBV birth-dose vaccination coverage below recommended thresholds [4]. Further, even in countries with improving aggregate indicators, within-country inequities persist, with urban, educated, and formally employed women benefiting disproportionately from integrated services while rural and marginalised communities remain underserved [1,4]. These patterns collectively establish the urgent need for a rigorous synthesis of integrated service delivery models that addresses all three infections simultaneously, across diverse settings.

The purpose of this systematic review and meta-analysis was to identify and synthesise evidence on integrated antenatal service delivery models addressing HIV, syphilis, and HBV simultaneously within maternal health services. The review specifically examined model characteristics, screening uptake, treatment and follow-up outcomes, implementation barriers and facilitators, and cost-effectiveness evidence where available. The significance of this review lies in: (a) consolidating evidence across all three infections simultaneously rather than treating them separately; (b) comparing integrated service delivery models across different income settings; (c) identifying barriers and facilitators affecting implementation; (d) examining whether screening translates into treatment, follow-up, and infant outcomes; and (e) clarifying evidence gaps for future research, policy, and programme design.

## 2. Materials and Methods

### 2.1. Protocol Registration, Reporting Standards, and Study Design

The protocol for this review was registered with PROSPERO (CRD420261342186) and conducted according to the Preferred Reporting Items for Systematic Reviews and Meta-Analyses (PRISMA 2020) guidelines (Supplementary Table S1) [13]. The review process involved conducting a literature search, screening studies, assessing eligibility, extracting data, and synthesising the results.

### 2.2. Eligibility Criteria

Studies were eligible for inclusion if they evaluated integrated service delivery approaches addressing all three infections targeted in the triple elimination initiative namely HIV, syphilis, and HBV, simultaneously within maternal or antenatal health services. Studies addressing only one or two of the three target infections were not eligible, as the review is specifically focused on models that integrate all three components of the WHO triple elimination initiative. Eligible studies examined interventions or programmes implemented within maternal health systems, including ANC, PMTCT services, maternal and child health programmes, or point-of-care screening initiatives delivered during pregnancy or the perinatal period. The review included empirical research reporting data from observational studies, programme evaluations, implementation studies, cross-sectional studies, cohort studies, pilot or intervention studies, and modelling studies that examined integrated screening, treatment, or prevention strategies for all three target infections. Eligible publications were restricted to studies published between 1 January 2007 and 10 January 2026 and written in English.

Studies were excluded if they focused on fewer than all three target infections without integrating services for all three simultaneously; evaluated diagnostic test accuracy without describing a service delivery model; consisted of editorials, commentaries, letters, narrative reviews, or other non-empirical reports; reported only planned interventions without empirical outcome data (i.e., study protocols); focused exclusively on non-maternal populations; or lacked sufficient information on the service delivery model.

The rationale for requiring coverage of all three infections is that the review question is specifically about integrated triple elimination service models. Studies integrating only two infections however valuable they are as evidence for individual components, do not constitute the integrated model described in the WHO triple elimination framework. Such studies are cited in the discussion where contextually relevant but are not included in the formal synthesis.

### 2.3. Information Sources

A systematic literature search was conducted in four databases: PubMed, Scopus, Web of Science, and Dimensions, for studies published between January 2007 and January 2026.

### 2.4. Search Strategy

The core PubMed search syntax was: (HIV OR “human immunodeficiency virus”) AND (syphilis OR “Treponema pallidum”) AND (“hepatitis B” OR HBV) AND (pregnancy OR antenatal OR prenatal OR maternal OR newborn OR infant) AND (integrated OR “service delivery” OR “point-of-care” OR “same-day” OR PMTCT OR EMTCT OR “triple elimination”). Equivalent search adaptations were developed for all databases. The complete search strategies are provided in Supplementary Table S2.

### 2.5. Selection Process

**Figure 1:**
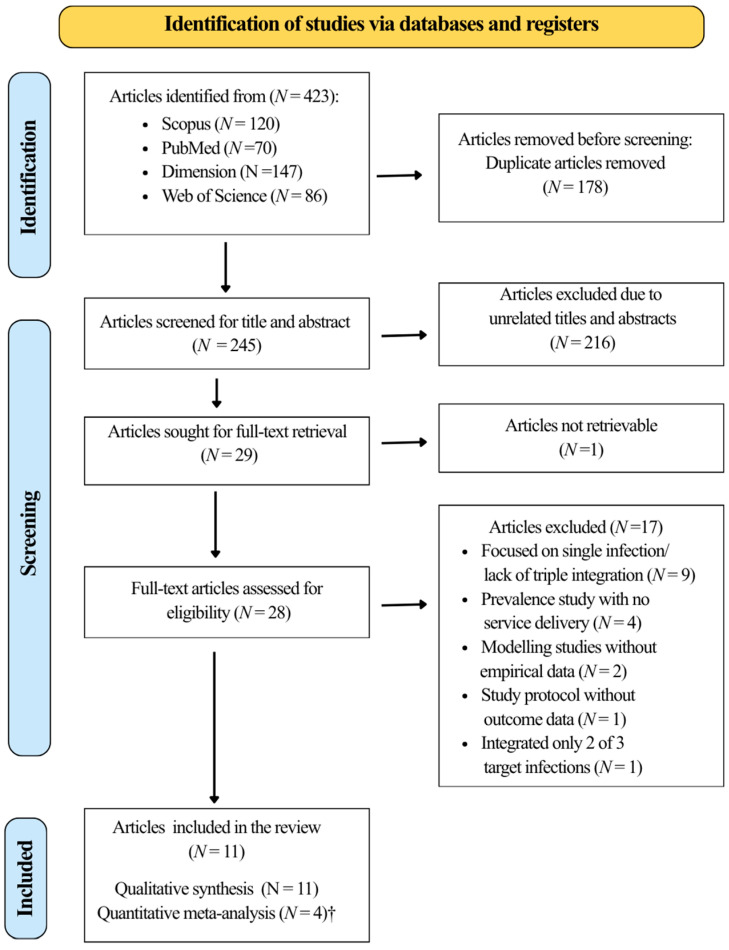
PRISMA 2020 flow diagram. † Studies contributing to quantitative meta-analysis (random-effects, DerSimonian–Laird estimator): Shan et al. [23], Nguyen et al. [24], Pai et al. [25], Martin et al. [26].

All records were imported into Rayyan [14], and duplicates were removed. Two reviewers (VAA, QESA) independently screened all titles, abstracts, and full-text articles. Disagreements were resolved by discussion or referral to a third reviewer (SB). A full list of excluded studies and reasons for exclusion is in Supplementary Table S3 [15,16,17,18,19,20,21,22]. Studies excluded at the full-text stage for addressing fewer than all three target infections are specifically noted in Supplementary Table S3 with this reason. The study selection process is summarised in the PRISMA flow diagram (Figure 1).

### 2.6. Data Collection Process

Data extraction was performed using an Excel-based structured data extraction template. Two reviewers (SJ, SB) independently extracted data and cross-checked for accuracy.

### 2.7. Data Items

Primary outcomes included triple testing uptake, ANC screening coverage, treatment uptake among infected mothers, MTCT rates, infant infection outcomes, and cost-effectiveness indicators. For each included study, Supplementary Table S5 specifies the exact combination of infections integrated; the type of intervention and service delivery platform; and programme components including diagnostics, treatment, HBV birth-dose vaccination, and infant follow-up. Implementation outcomes including acceptability and feasibility were also extracted when reported.

### 2.8. Study Risk of Bias Assessment

Risk of bias was assessed using ROBINS-I for observational studies, the JBI critical appraisal checklist for cohort and pre-post studies, and MMAT for mixed-methods studies (Supplementary Table S4). Modelling studies were evaluated qualitatively based on the transparency of model assumptions. These risk-of-bias assessments shaped the interpretation of findings throughout as studies with a higher risk of bias (small pilots and single-centre cross-sectional studies) received more cautious interpretation, while programme evaluations and national datasets were considered to provide stronger implementation evidence despite the absence of causal inference designs.

### 2.9. Effect Measures

Effect measures included proportions describing screening uptake and treatment coverage, MTCT rates, averted cases in modelling studies, and cost-effectiveness ratios where reported.

### 2.10. Data Synthesis

Studies were grouped according to the type of integrated service delivery model. Narrative synthesis was performed for all included studies. For quantitative synthesis, studies reporting numerator/denominator data for triple testing uptake were identified. A meta-analysis of proportions was conducted for the four studies providing these data [23,24,25,26]. Statistical analysis was performed using an online meta-analysis software available at (https://metaanalysisonline.com/) (accessed on 6 April 2026) [27]. A random-effects model was applied using the DerSimonian–Laird estimator. Proportions were logit-transformed prior to pooling to stabilise variance and improve distributional properties, with back-transformation applied for presentation. No continuity correction was required, as no study reported zero events. Statistical heterogeneity was assessed using the I^2^ statistic. Potential sources of heterogeneity were explored qualitatively. Formal sensitivity analyses were not conducted given the small number of contributing studies.

### 2.11. Reporting Bias Assessment

A formal funnel plot or statistical assessment of reporting bias was not conducted because only four studies contributed to the quantitative synthesis, which limits the reliability of such analyses. However, the risk of publication bias cannot be excluded as studies reporting high testing uptake are more likely to be published, and this may inflate the pooled estimate. This limitation is acknowledged in the interpretation of findings.

### 2.12. Certainty of Evidence

A formal GRADE assessment was not performed due to substantial heterogeneity in study designs, outcome measures, and reporting formats [28]. The overall certainty of evidence was interpreted qualitatively, considering study design, sample size, risk-of-bias assessments, and consistency of findings.

## 3. Results

### 3.1. Characteristics of Included Studies

Eleven studies were included in this review, and their characteristics are summarised in Supplementary Table S5 [7,8,9,10,23,24,25,26,29,30,31]. Two studies identified during screening were excluded at the full-text stage for not meeting the criterion of empirical results from an integrated service model addressing all three target infections simultaneously: a study from Mozambique [32] that integrated HIV and HBV services without syphilis, and a protocol study from Burkina Faso and The Gambia [33] without empirical outcome data. Both are cited in the discussion for contextual evidence. The included studies were conducted across multiple geographical regions and income settings. Geographically, studies were distributed across Asia, Africa, Europe, and Latin America. In Asia, studies were conducted in China [9,23], Vietnam [24], India [25], Nepal [8], and Indonesia [29]. African studies were conducted in Zimbabwe [26] and Kenya [30]. Additional evidence came from Europe (the Netherlands [10]) and Latin America (Guatemala [31]). Two modelling studies used programme data from Cambodia [7] and Nepal [8].

Of the 11 included studies, nine were conducted in low- or lower-middle-income countries, reflecting the focus of triple elimination initiatives in resource-constrained health systems: Vietnam, India, Nepal, Indonesia, Zimbabwe, Kenya, Guatemala, and Cambodia (modelled) and Nepal (modelled). Two studies were conducted in upper-middle-income China [9,23], and one in a high-income country, the Netherlands [10].

The included studies used a variety of research designs such as observational national programme evaluations; cross-sectional or facility-based pilot studies [27,33]; pre- and post-intervention implementation studies [24]; mixed-methods process evaluations [26]; trend analyses using programme surveillance data [25,28]; microsimulation modelling studies [7,8]; and implementation reports [31]. These study types carry different evidentiary weights. Modelling studies provide projections rather than observed effects; pilot studies may not generalise to routine programme conditions; and implementation reports typically lack full denominators. These distinctions are considered throughout the narrative synthesis.

Sample sizes varied substantially. Facility-level pilot and implementation studies typically enrolled approximately 600–1100 pregnant women, as seen in India [25], Zimbabwe [26], and Kenya [30]. National programme evaluations in China [9,23] and the Netherlands [10] covered hundreds of thousands to millions of pregnancies.

### 3.2. Quantitative Synthesis of Triple-Testing Uptake

**Figure 2:**
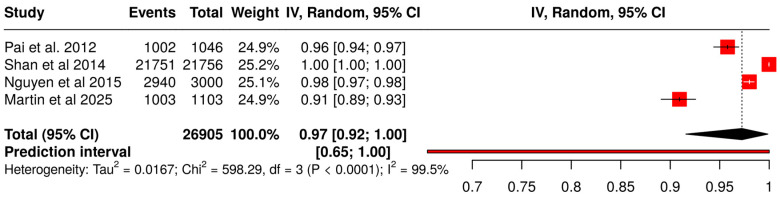
Forest plot showing pooled uptake of integrated triple testing among pregnant women across four studies. Pooled proportion: 0.97 (95% CI 0.92–1.00), random-effects model, DerSimonian–Laird estimator [23,24,25,26].

Four studies reported the number of women who received all three tests (HIV, syphilis, and HBV) during ANC and the total number approached which enabled calculation of triple-testing uptake: Shan et al. (Dehong, China) [23], Nguyen et al. (Vietnam) [24], Martin et al. (Zimbabwe) [26], and Pai et al. (India) [25]. A random-effects meta-analysis (DerSimonian–Laird estimator, logit transformation) showed a pooled triple-testing uptake of 97% (95% CI 92–100%) (Figure 2). Heterogeneity was high (I^2^ = 99.5%), reflecting variability in settings and programme maturity. The prediction interval was wide (0.65–1.00). Given this extreme heterogeneity, the pooled estimate should be interpreted cautiously as reflecting the central tendency across these four diverse programmes rather than a generalisable benchmark. Formal subgroup analyses were not feasible given the small number of contributing studies. Qualitative exploration of heterogeneity sources is presented in the discussion.

### 3.3. Integration Models and Implementation Insights

**Figure 3:**
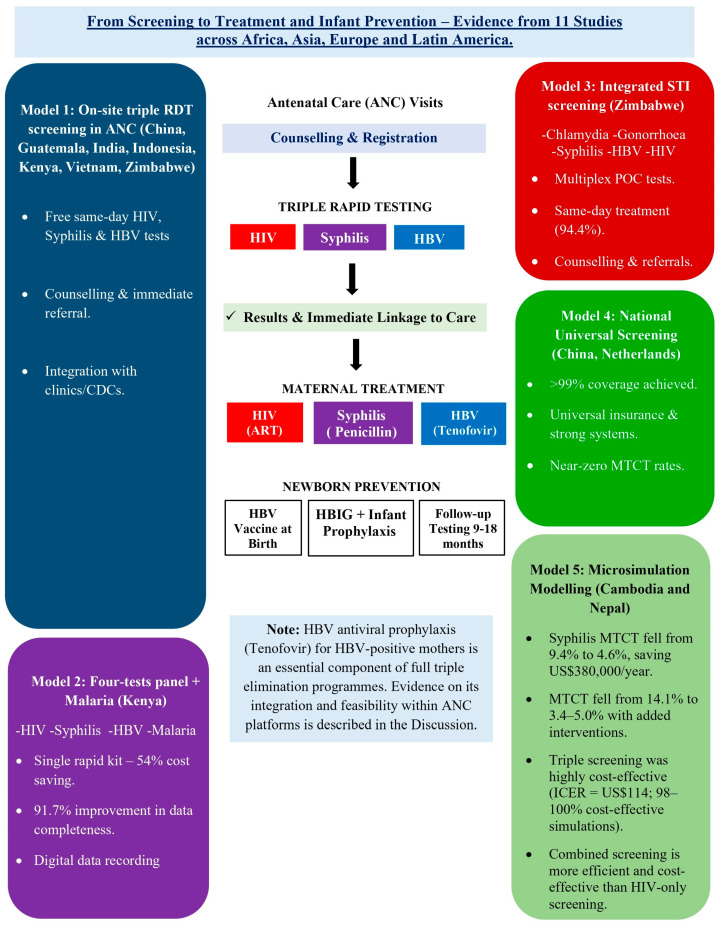
Integrated service delivery models for triple elimination of HIV, syphilis, and HBV in antenatal care. Evidence from 11 included studies across Asia, Africa, Europe, and Latin America. Five models are shown [7,8,9,10,23,24,25,26,29,30,31]. The care cascade (counselling → triple testing → linkage → treatment → newborn prevention → follow-up) is illustrated in the centre panel. Evidence for HBV antiviral integration feasibility is described in the Discussion [32]. ANC = antenatal care; CDC = Centre for Disease Control; HBIG = hepatitis B immunoglobulin; ICER = Incremental Cost-Effectiveness Ratio; MTCT = mother-to-child transmission; POC = point-of-care; RDT = rapid diagnostic test.

Five integration models were observed from the 11 included studies (Figure 3). The first model, on-site triple RDT packages, was implemented in China, Vietnam, India, Zimbabwe, Kenya, Indonesia, and Guatemala, combining HIV, syphilis, and HBV tests during the same ANC visit [9,25,26,27,28,29,32,33]. These models typically involved midwives or nurses using RDTs, followed by counselling and immediate referral. Free services and same-day results were key facilitators. In China’s national programme, testing coverage exceeded 99% and HIV MTCT was reduced [9]. In Dehong, China, testing coverage for all three infections reached 99.9% by 2013, with HIV MTCT of 2.28–3.00% [23]. In Zimbabwe, 91% of eligible women accepted complete triple-plus STI screening, with 98.4% of positive cases receiving same-day treatment, though health-worker workload and supply-chain reliability were challenges [26]. In India, 96% of women completed simultaneous triple POC testing [25].

The second model involved expanded PMTCT packages with malaria integration, implemented in Kenya, where a four-test panel (HIV, syphilis, HBV, and malaria) reduced costs by 54% and improved data completeness by 91.7% [30]. The third model, integrated point-of-care screening including curable STIs, was implemented in Zimbabwe, where testing expanded to include chlamydia and gonorrhoea alongside HIV, syphilis, and HBV [26]. The fourth model involved national universal screening programmes, as exemplified in the Netherlands and China, achieving screening coverage above 99% and near-zero MTCT rates [10,23]. The fifth model comprised microsimulation modelling studies from Cambodia [7] and Nepal [8], which demonstrated the cost-effectiveness of triple screening compared with dual or single-infection approaches.

### 3.4. Implementation Barriers and Facilitators

**Figure 4:**
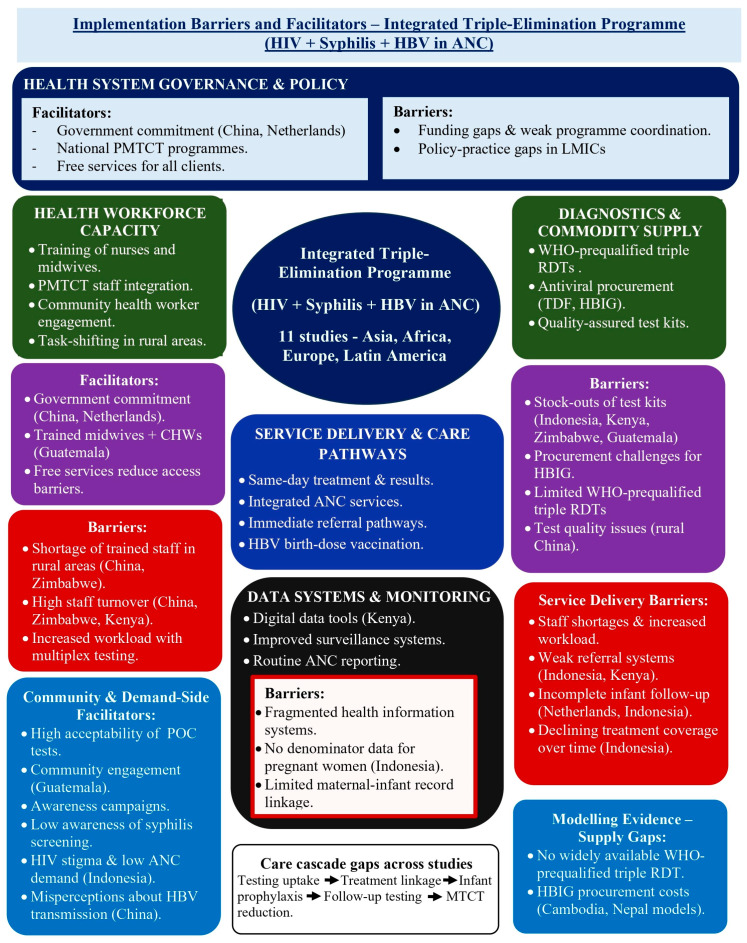
Implementation barriers and facilitators for the integrated triple-elimination programme (HIV + Syphilis + HBV) in antenatal care. Findings from 11 included studies across Asia, Africa, Europe, and Latin America. ANC = antenatal care; CHW = community health worker; HBIG = hepatitis B immunoglobulin; MTCT = mother-to-child transmission; POC = point-of-care; RDT = rapid diagnostic test; TDF = tenofovir disoproxil fumarate.

Barriers and facilitators are drawn from the 11 included studies (Figure 4). Facilitators included strong political commitment in China and the Netherlands [9,10,23], donor- or government-funded programmes enabling free testing and treatment, use of RDTs for same-day results, integration with existing maternal–child health systems, and community engagement strategies in Guatemala [31]. Barriers frequently reported included stock-outs of diagnostic test kits and medications in Indonesia, Kenya, Zimbabwe, and Guatemala [28,29,32,33]. Shortages and high turnover of trained staff were reported in China and Zimbabwe [9,26]. Other system-level challenges included incomplete data systems and limited follow-up for exposed infants in Indonesia and the Netherlands [10,29]. In Indonesia, limited awareness and stigma surrounding STIs contributed to low uptake of syphilis screening [29]. Modelling studies highlighted the lack of WHO-prequalified triple RDTs and procurement challenges with HBIG [7,8].

## 4. Discussion

This review synthesised evidence from 11 studies evaluating antenatal service models that integrate HIV, syphilis, and HBV testing and treatment simultaneously. The findings demonstrate that integrated programmes can achieve very high coverage of triple testing and markedly reduce vertical transmission when implemented at scale. National programmes in China and the Netherlands achieved testing coverage exceeding 99% and reduced HIV MTCT to below 3% [9,10], and a regional programme in China’s Dehong prefecture reported similarly impressive results [23]. At the other end of the spectrum, small pilots in Vietnam, India, and Zimbabwe attained high acceptance but faced supply-chain interruptions, variable linkage to treatment, and modest sample sizes [27,28,32]. These contrasts highlight both the potential and the fragility of integration, in which performance depends on scale, infrastructure, and continuous support.

The success of integrated services must be interpreted against a backdrop of persistent global burden. Cross-sectional surveys show that even where antenatal testing is available, the prevalence of co-infection remains substantial. In Ethiopia’s Amhara region, HIV, HBV, and syphilis were detected in 7.6%, 4.5%, and 3.3% of pregnant women, respectively [2]. Many women in Taiyuan, China, were first diagnosed with HBV during pregnancy and lacked knowledge about viral markers [34]. Brazilian surveillance data reveal a sharp increase in congenital syphilis from 4.7 to 27.1 per 1000 births between 2011 and 2021 and declining birth-dose HBV vaccination coverage [11]. Such trends justify expanding the focus to triple elimination.

Differences across income settings illuminate what it takes to achieve triple elimination. High-income settings like the Netherlands benefit from universal insurance, strong laboratory networks, and robust civil registration systems, enabling >99% screening [10]. Upper-middle-income China scaled integrated testing nationwide through county-level maternal and child health clinics, supported by the Centre for Disease Control and the central government [9]. By contrast, many low-income countries lack such foundations. Indonesia’s Bandung programme increased HIV screening coverage from 26.5% to 54.2%, but treatment coverage for HIV-positive women dropped from 87.5% to 59.5% over three years [29]. A secondary hospital in North Bali achieved 98.5% testing coverage but only 6.6% of HBV-positive mothers received antiviral prophylaxis [35]. Such gaps reflect limited capacity in LMICs and emphasise that triple elimination depends not only on screening but also on the full care cascade.

Two studies identified during this review provide important contextual evidence. A pilot study in Mozambique integrated HBV screening (HBsAg RDT) with tenofovir prophylaxis and HBV birth-dose vaccination into existing HIV PMTCT services [32]. Though syphilis was not integrated, this pilot achieved 83.4% HBV birth-dose vaccination and HBsAg positivity of only 0.7% in infants at nine months and demonstrated the feasibility and clinical value of adding antiviral prophylaxis to PMTCT platforms, a component that will be essential for future triple elimination programmes. A protocol study from Burkina Faso and The Gambia described a simplified integrated strategy for simultaneous HIV–syphilis–HBV elimination in West Africa [33], a geographically under-represented region and a forthcoming source of evidence on triple elimination. Results from the TRI-MOM project will be important for future systematic reviews.

Across studies, common barriers included stock-outs of rapid test kits, benzathine penicillin, tenofovir, and hepatitis B immunoglobulin, which impeded continuity in Indonesia, Zimbabwe, and Kenya [32,33]. High turnover and uneven distribution of trained nurses hindered programme coverage in rural China, Kenya, and Guatemala. Qualitative research among migrant women in China highlighted stigma, lack of trust, and logistical barriers to PMTCT uptake [12]. In South Africa, a pilot of point-of-care HBV screening found that universal infant vaccination is feasible but logistically challenging [36]. In Brazil, the rapid rise in congenital syphilis and declining vaccination coverage were attributed to supply-chain gaps [11].

It is important to note that the South Africa study [36] and the Ghana implementation research study [37] were excluded because the South Africa study focused on HBV screening without a fully integrated triple service model, and the Ghana study was a rapid qualitative study without empirical service delivery outcome data but they provide contextual information to understand implementation barriers and facilitators.

Implementation research is beginning to unpack the complex organisational and policy context required for triple elimination. A qualitative study in Ghana applied the Consolidated Framework for Implementation Research and identified eight key barriers, including policy gaps, cost burdens, and limited leadership engagement, and four facilitators including perceived evidence strength [37]. Cross-country comparisons indicate that community engagement and education are crucial. In Guatemala, community health workers and midwives in outreach teams raised antenatal coverage to 99.6% [31], whereas in Taiyuan, misperceptions about HBV transmission persisted despite frequent hospital contact [34].

Despite high pooled uptake of triple testing (97%), the meta-analysis revealed extreme heterogeneity (I^2^ ≈ 99.5%). This is attributable to several factors. For instance, the four contributing studies span single-facility pilots (India, Zimbabwe) and multi-facility regional programmes (China, Vietnam); they differ in income setting, programme maturity, sample size, and funding modality and near-ceiling proportions in some studies (e.g., 98–99% in Vietnam and Dehong, China) create limited variance for the estimator. Given this heterogeneity, the pooled estimate of 97% should not be taken as a reliable benchmark for new programmes. However, the prediction interval of 65–100% better reflects the realistic range. Formal subgroup analyses by income level, programme type, or health-system tier were not possible given only four contributing studies. Future comparative designs such as cluster-randomised trials or stepped-wedge evaluations are needed to identify which integration components yield the greatest incremental benefit.

There are several policy implications. First, national programmes should adopt integrated antenatal screening for all three infections as part of universal health coverage and align with WHO’s validation metrics [6]. Second, governments and donors must address commodity security, including procurement of quality-assured triple RDTs, benzathine penicillin, antivirals, and HBIG [7]. Third, workforce capacity must be strengthened through continuous training, mentoring, and task-shifting. Fourth, birth-dose HBV vaccination should be universal and timely, building on evidence that high coverage is achievable when integrated with PMTCT [32]. Finally, community engagement must address misperceptions about HBV and tackle HIV stigma [34].

Future research must go beyond feasibility studies to evaluate integration models across diverse settings and examine long-term outcomes, including infant seroconversion, maternal retention in care, and cost-effectiveness. Results from the TRI-MOM project in Burkina Faso and The Gambia [33] will be particularly valuable for the West African context, where evidence is currently absent. Implementation scientists should also explore how to embed triple testing into broader maternal health and child immunisation platforms.

### Limitations

This review has several limitations. First, only four studies contributed data to the quantitative meta-analysis, and heterogeneity was extreme (I^2^ = 99.5%), limiting the reliability of the pooled estimate. Second, the majority of included studies are observational or pilot in design, providing limited causal inference; two studies are modelling studies that provide projections rather than observed outcomes. Third, non-English publications were excluded, which may have resulted in the omission of relevant studies from non-Anglophone settings. Fourth, the small number of studies precluded formal statistical testing of publication bias, and selective reporting of positive outcomes cannot be excluded; studies reporting high uptake are more likely to be published. Fifth, the diverse outcome reporting across studies prevented systematic meta-analysis of treatment uptake, infant outcomes, or cost-effectiveness, and the review relies heavily on narrative synthesis for those endpoints. Sixth, the inclusion criterion requiring all three infections to be integrated simultaneously, while methodologically sound, means that important partial-integration evidence (such as the Mozambique HBV antiviral pilot) is excluded from the formal synthesis. These limitations caution against overstating the certainty of the evidence base.

## 5. Conclusions

Evidence from 11 studies across low-, middle-, and high-income countries demonstrates that integrated antenatal service delivery models for simultaneous triple EMTCT of HIV, syphilis, and HBV appear feasible and can achieve high testing coverage, particularly in well-supported programmes. However, the evidence base remains predominantly observational and several included studies are pilot or modelling studies that do not provide direct proof of programme-level effectiveness. Conclusions should therefore be interpreted accordingly. Screening uptake, while high when measured, is only one step in the care cascade. Future research must evaluate the full cascade which includes treatment linkage, maternal retention, infant prophylaxis, HBV birth-dose vaccination, infant testing, and reduction in actual transmission rates. Future programmes should prioritise the procurement of quality diagnostics, continuous workforce training, community engagement, robust data systems, and integrated follow-up for mothers and infants. Modelling studies show that triple screening is cost-effective and can avert substantial morbidity and mortality. Adoption of integrated models at scale, informed by ongoing studies such as TRI-MOM, will accelerate progress towards global EMTCT targets.

## Data Availability

No new data were created or analysed in this study.

## Supplementary Materials

The following supporting information can be downloaded at: https://www.mdpi.com/article/10.3390/healthcare14121625/s1, Table S1: PRISMA Checklist; Table S2: Search queries; Table S3: List of articles excluded [3,4,5,15,16,17,18,19,20,21,22,32,33,34]; Table S4: Risk of bias assessment; Table S5: Detailed data extraction for included studies.

## Abbreviations

The following abbreviations are used in this manuscript:

HIV	Human immunodeficiency virus
HBV	Hepatitis B Virus
MTCT	Mother-to-child transmission
HBeAg	Hepatitis B e-antigen
WHO	World Health Organization
DALY	Disability-adjusted life year
EMTCT	Eliminating mother-to-child transmission
ANC	Antenatal care
PMTCT	Prevention of mother-to-child transmission
PROSPERO	International Prospective Register of Systematic Reviews
PRISMA	Preferred Reporting Items for Systematic Reviews and Meta-Analyses
JBI	Joanna Briggs Institute
MMAT	Mixed Methods Appraisal Tool
GRADE	Grading of Recommendations Assessment, Development, and Evaluation
HBIG	Hepatitis B immunoglobulin
STIs	Sexually transmitted infections
