# Supplementary material for: Integrated Service Delivery Models for Triple Elimination of Mother to Child Transmission of Human Immunodeficiency Virus, Syphilis, and Hepatitis B Virus: A Global Systematic Review and Meta-Analysis
Source: Healthcare (Basel). 2026 Jun 9;14(12):1625. doi: 10.3390/healthcare14121625 (PMC13299941; doi:10.3390/healthcare14121625)
Supplement: Supplementary file 1 [file healthcare-14-01625-s001.zip › Supplementary_Tables_S2_S3_S4_Updated.pdf]

Supplementary Tables S2

Supplementary Table S2. Database Search Strategies

Search conducted across four databases: PubMed, Scopus, Web of Science, and Dimensions. Date range: 1 January 2007 to 10 January 2026. Language filter: English. Complete strategies shown below. Total records identified: N = 423.

Database	Search Strategy	Results (N)
**PubMed**	(("HIV"[Title/Abstract] OR "human immunodeficiency virus"[Title/Abstract]) AND (syphilis[Title/Abstract] OR "Treponema pallidum"[Title/Abstract]) AND ("hepatitis B"[Title/Abstract] OR HBV[Title/Abstract]) AND (pregnan*[Title/Abstract] OR antenatal[Title/Abstract] OR prenatal[Title/Abstract] OR maternal[Title/Abstract] OR perinatal[Title/Abstract] OR newborn*[Title/Abstract] OR infant*[Title/Abstract]) AND (integrat*[Title/Abstract] OR "service delivery"[Title/Abstract] OR "one-stop"[Title/Abstract] OR "point-of-care"[Title/Abstract] OR bundled[Title/Abstract] OR cascade[Title/Abstract] OR linkage[Title/Abstract] OR "same-day"[Title/Abstract] OR "maternal and child health"[Title/Abstract] OR PMTCT[Title/Abstract] OR EMTCT[Title/Abstract] OR "triple elimination"[Title/Abstract]))	70
**Scopus**	TITLE-ABS-KEY((HIV OR "human immunodeficiency virus") AND (syphilis OR "Treponema pallidum") AND ("hepatitis B" OR HBV) AND (pregnan* OR antenatal OR prenatal OR maternal OR perinatal OR newborn* OR infant*) AND (integrat* OR "service delivery" OR "one-stop" OR "point-of-care" OR bundled OR cascade OR linkage OR "same-day" OR "maternal and child health" OR PMTCT OR EMTCT OR "triple elimination"))	120
**Web of Science**	("HIV" OR "human immunodeficiency virus") AND (syphilis OR "Treponema pallidum") AND ("hepatitis B" OR HBV) AND (pregnancy OR antenatal OR prenatal OR maternal OR newborn OR infant) AND (integrated OR "service delivery" OR "point-of-care" OR "same-day" OR PMTCT OR EMTCT OR "triple elimination")	86
**Dimensions**	TS=((HIV OR "human immunodeficiency virus") AND (syphilis OR "Treponema pallidum") AND ("hepatitis B" OR HBV) AND (pregnan* OR antenatal OR prenatal OR maternal OR perinatal OR newborn* OR infant*) AND (integrat* OR "service delivery" OR "one-stop" OR "point-of-care" OR bundled OR cascade OR linkage OR "same-day" OR "maternal and child health" OR PMTCT OR EMTCT OR "triple elimination"))	147
**Total**	**All four databases — date range: 1 January 2007 to 10 January 2026; language filter: English**	**423**

Supplementary Table S3. List of Articles and Reasons for Exclusion

Seventeen articles were excluded at full-text stage (*N = 14*). All excluded studies are listed below with full citation details and exclusion reasons.

No.	Author(s) & Year	Title	Country	Reason for Exclusion
1.	Njau et al., 2025 [15]	*Prevalence and associated factors for HIV, HBV, and syphilis coinfections among pregnant women attending antenatal care in Tanzania*	Tanzania	Cross-sectional prevalence study; no integrated service delivery component.
2.	Takahashi et al., 2024 [16]	*Barriers and facilitators for preventing MTCT of Trypanosoma cruzi and hepatitis B in the Gran Chaco region*	Bolivia & Argentina	Focused on Trypanosoma cruzi and HBV; did not address HIV; integration of two infections only, neither being the three target infections.
3.	Wulandari et al., 2024 [17]	*Challenges to integrating programs for the elimination of MTCT of HIV, syphilis and hepatitis B into antenatal care: Experiences from Indonesia*	Indonesia	Qualitative study reporting barriers; does not provide quantitative outcomes on screening, treatment, or MTCT; excluded at data-extraction stage.
4.	Bell et al., 2023[5]	*Progress toward triple elimination of MTCT of HIV, hepatitis B and syphilis in Pacific Island Countries and Territories: a systematic review*	Pacific region	Scoping review describing regional progress; no specific service delivery model or extractable programme outcome data.
5.	Lian et al., 2025 [3]	*Global burden of HIV, syphilis, and HBV infection among women of childbearing age and children under five: based on the Global Burden of Diseases Study 2021*	Global	Burden estimation study; not a service delivery or implementation study.
6.	Woodring et al., 2017 [18]	*Integrated HIV, hepatitis B and syphilis screening and treatment through the reproductive, maternal, newborn and child health platform to reach global*	Western Pacific	Commentary on integration strategies; lacks extractable programme data.

No.	Author(s) & Year	Title	Country	Reason for Exclusion
		elimination targets		
7.	Kakkar & Boucoiran, 2018[19]	*The Women and Children's Infectious Diseases Center: An integrated approach to congenital infectious diseases*	—	Commentary; no empirical programme data.
8.	Martin et al., 2025 [20]	*The contribution of PrEP programmes to triple elimination efforts: a cross-sectional study of status and opportunities*	South Africa	Focused on PrEP and a different population; did not evaluate integrated antenatal triple elimination service delivery.
9.	Armini et al., 2024 [21]	*Patient perspective on the elimination of MTCT of HIV, syphilis and hepatitis B in Bali, Indonesia*	Indonesia	Qualitative study exploring patient perceptions; no quantitative service delivery outcomes.
10.	Cohn et al., 2021 [4]	*Eliminating MTCT of HIV, syphilis and hepatitis B in sub-Saharan Africa*	Africa	Narrative review summarising policies; not an empirical study.
11.	Liu et al., 2025 [34]	*Prevalence and associated factors with low birth weight among HIV-exposed infants in Hubei, China*	China	Cohort study of birth weight among HIV-exposed infants; not an integrated triple service study.
12.	Mo et al., 2023 [22]	*Treatment of maternal syphilis for preventing congenital syphilis: an observational study of adherence to treatment recommendation in Suzhou, China, 2019–2021*	China	Single infection (syphilis only); full text inaccessible .
13.	*Loarec et al., 2022* [32]	*Prevention of mother-to-child transmission of hepatitis B virus in antenatal care and maternity services, Mozambique*	Mozambique	*Integrated HIV and HBV only; syphilis not included. Does not meet revised inclusion criterion requiring simultaneous integration of all three target infections (HIV, syphilis, and HBV). Cited in Discussion as contextual*

No.	Author(s) & Year	Title	Country	Reason for Exclusion
				evidence on HBV antiviral integration feasibility [Ref. 34].
14.	*Vo-Quang et al., 2026* [33]	*Towards triple elimination of HIV, syphilis and HBV mother-to-child transmission: Protocol of a simplified and integrated strategy in Burkina Faso and The Gambia (TRI-MOM)*	Burkina Faso & Gambia	*Study protocol without empirical outcome data. Evidence synthesis requires empirical results. Cited in Discussion as forthcoming evidence from West Africa [Ref. 35].*

Supplementary Table S4. Risk of Bias Assessment

Risk of bias was assessed using ROBINS-I for observational studies, the JBI critical appraisal checklist for cohort and cross-sectional studies, and MMAT for mixed-methods studies. Modelling studies were evaluated qualitatively.

Study	Study Design	Risk-of-Bias Tool	Key Domains Assessed	Overall Judgement
**Wang et al. 2015 [9]**	Observational national programme evaluation	ROBINS-I	Confounding: moderate (no control group); Selection: low (national programme); Classification of intervention: low; Deviations from intended interventions: low; Missing data: low; Outcome measurement: moderate (registry data). Higher risk of bias assessments interpreted cautiously in narrative synthesis.	**Moderate**
**Zhang et al. 2019 [7]**	Decision-tree cost-effectiveness modelling	Not applicable (modelling)	Transparent use of empirical parameters and sensitivity analyses; assumptions documented; projections only, not observed outcomes.	**Low (modelling)**
**Nguyen et al. 2021 [24]**	Pre/post observational implementation study	ROBINS-I	Confounding: serious (no control group); Selection: moderate; Measurement: low; Missing data: unclear. Cautious interpretation applied.	**Serious**
**Martin et al. 2025 [26]**	Mixed-methods process evaluation	MMAT & ROBINS-I	Confounding: moderate; Selection: moderate; Measurement: low; Response bias: moderate. Caution applied to qualitative components.	**Moderate**
**Ochwoto et al. 2024 [30]**	Cross-sectional pilot study	JBI cross-sectional checklist	Sampling: moderate; Measurement: low; Response rate: not reported; Confounding: moderate. Small single-setting pilot — limited generalisability.	**Moderate**
**Visser et al.**	National registry	ROBINS-I	Confounding: low; Selection: low (universal	**Low**

Study	Study Design	Risk-of-Bias Tool	Key Domains Assessed	Overall Judgement
**2019 [10]**	evaluation		coverage); Outcome measurement: low; Reporting bias: low. National programme evaluation — stronger implementation evidence.	
**Sabin et al. 2025 [8]**	Microsimulation modelling	Not applicable (modelling)	Uses empirical inputs with sensitivity analyses; transparency reported; projections only, not observed outcomes.	**Low (modelling)**
**Pai et al. 2012 [25]**	Prospective observational pilot	ROBINS-I	Confounding: moderate; Selection: moderate; Measurement: low; Sample size: small. Cautious interpretation applied to generalised conclusions.	**Moderate**
**Shan et al. 2014 [23]**	Observational surveillance evaluation	ROBINS-I	Confounding: moderate (no control); Selection: low (population-level); Outcome measurement: moderate (registry and clinical records). Interpreted as stronger implementation evidence due to scale.	**Moderate**
**Azhali et al. 2023 [29]**	Trend analysis, programme surveillance data	ROBINS-I	Confounding: serious (no comparator, declining denominators); Missing data: serious; Outcome measurement: moderate. High risk — interpreted with caution.	**Serious**
**Smith et al. 2015 [31]**	Implementation report	JBI-insufficient data	Very limited data available unable to fully assess risk of bias across domains.	**Unclear**
ROB interpretation note: Risk-of-bias assessments shaped narrative synthesis throughout. Studies assessed as Serious risk were interpreted with substantial caution. Moderate-risk studies (mostly pilot and single-centre designs) informed feasibility findings but were not used to draw strong effectiveness conclusions. Low-risk national programme evaluations (Visser, Wang) and modelling studies (Zhang, Sabin) provided the strongest implementation and cost-effectiveness evidence respectively.				

ROBINS-I = Risk Of Bias In Non-randomised Studies of Interventions; JBI = Joanna Briggs Institute; MMAT = Mixed Methods Appraisal Tool. ROB = Risk of bias.
